# Ureteroscopy Is Equally Efficient and Safe in Obese and Morbidly Obese Patients: A Systematic Review and Meta-Analysis

**DOI:** 10.3389/fsurg.2022.736641

**Published:** 2022-02-18

**Authors:** Wei Wang, XiaoShuai Gao, Liao Peng, Tao Jin

**Affiliations:** Department of Urology, Institute of Urology, West China Hospital, Sichuan University, Chengdu, China

**Keywords:** ureteroscopy, urolithiasis, obesity, meta-analysis, systematic review

## Abstract

**Background:**

Ureteroscopy (URS) has been established as an effective treatment for stones in obese patients (OP). However, recent studies found that the efficacy of the procedure may be lower in patients with higher body mass index (BMI). In the current study, we aim to determine if obesity might influence the effectiveness and safety of URS.

**Methods:**

In May 2021, a comprehensive search was conducted in the PubMed, EMBASE, Web of Science, Cochrane Library, and ClinicalTrials.gov to find eligible studies. Stone-free rate (SFR), operative time, length of stay, and complication rate were assessed utilizing RevMan 5.3.

**Results:**

Thirteen studies involving 4,583 normal-weight patients (NWP), 2,465 OP, and 291 morbidly OP (MOP) were included. Pooled results showed that statistically similar SFR existed between OP and NWP [odds ratio (*OR*): 1.09; 95% *CI*: 0.79, 1.52; *p* = 0.59], and between MOP and NWP (*OR*: 1.03; 95% *CI*: 0.46, 2.31; *p* = 0.95). The operation time was similar between OP and NWP [mean difference (*MD*): −2.27; 95% *CI*: −8.98, 4.43; *p* = 0.51], and between MOP and NWP (*MD*: 4.85; 95% *CI*: −5.78, 15.47; *p* = 0.37). In addition, no significant difference regarding length of stay existed between OP and NWP (*MD*: −0.07; 95% *CI*: −0.20, 0.07; *p* = 0.33), and between MOP and NWP (*MD*: −0.06; 95% *CI*: −0.25, 0.14; *p* = 0.58). Furthermore, we observed similar minor complication rate between OP and NWP (*OR*: 1.04; 95% *CI*: 0.81, 1.32; *p* = 0.78), and between MOP and NWP (*OR*: 1.29; 95% *CI*: 0.80, 2.08; *p* = 0.30). The differences concerning major complication rate between OP and NWP (*OR*: 0.97; 95% *CI*: 0.39, 2.43; *p* = 0.95), and between MOP and NWP (*OR*: 2.01; 95% *CI*: 0.55, 7.30; *p* = 0.29) were also not significant.

**Conclusions:**

Our study demonstrated that URS performed in MOP and OP appears to have the same efficacy and safety as well as in NWP group.

## Introduction

Currently, the worldwide epidemic, obesity, and overweight affect one-third population of the world (1). The obesity rate has increased in all ages, regardless of countries, regions, ethnicity, and socioeconomic status. Currently, the WHO gives a clear definition on normal weight: a body mass index (BMI) value <25 kg/m^2^; whereas a BMI value >30 kg/m^2^ is regarded as obese patients (OP), and a BMI value above 40 kg/m^2^ is morbidly OP (MOP) (1). Obesity impairs normal physiological functions of the body and substantially increases the risks for developing many medical problems, such as diabetes mellitus (2), angiocardiopathy (3), musculoskeletal diseases (4), and several kinds of cancers (5). Recently, many researches have demonstrated that obesity also raised the risks of urolithiasis (6–8). Possible mechanisms underlying the association are changes in renal acid/base handling (9), alterations in urine composition (8, 10), insulin resistance, and increased production of fatty acids (11).

Obesity poses a management challenge in terms of imaging, anesthesia, and surgical methods to urologists (12). Usually, urolithiasis is diagnosed by ultrasound and CT scan, but the increased thickness of fat tissue prevents acquiring a high-quality image, and some obese individuals might be unable to fit into the CT scanner due to excessive weight or abdominal circumference (12). Furthermore, anesthesia can induce several respiratory function alterations in the OP, such as the decrease in the functional residual capacity and respiratory compliance (13).

As for surgical options of the obese individuals with kidney stones, Extracorporeal Shock Wave Lithotripsy might not be a suitable procedure choice due to the farther skin-to-stone distance (12); for another treatment method, percutaneous nephrolithotomy, the standard prone position can increase the respiratory impairment and impedes venous blood flow in OP (14). All above-mentioned make ureteroscopy (URS) is the only feasible procedure. Recently, several studies have been published to evaluate the effectiveness of BMI on URS with controversial results (15–27). Some authors reported that stone-free rate (SFRs), complications, operation time, and length of stay were independent of BMI (15–23, 25–27), while Krambeck et al. showed the SFR of the URS procedure in obese individuals was lower compared with normal-weight patients (NWP) group (24). Therefore, we performed a meta-analysis, for the first time, to determine if obesity might influence the effectiveness and safety of URS with NWP controlled. We hope the results of the presented study could be helpful for urologists and their patients in decision-making process. This will definitely increase the chance to select the best method for our patients.

## Methods

### Search Strategy

**Figure 1:**
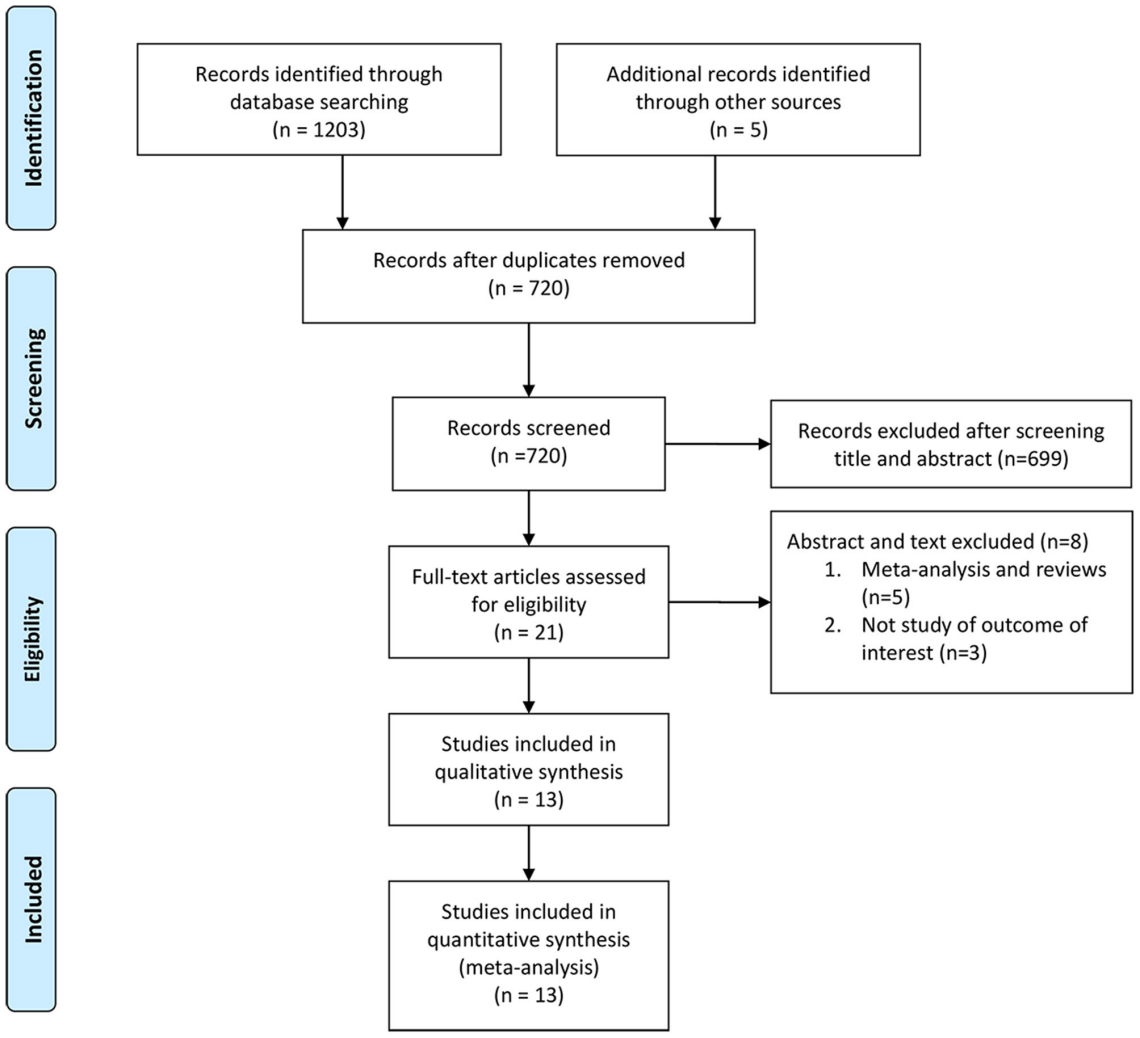
The flow chart showing study search and selection process.

A comprehensive search in the PubMed, EMBASE, Web of Science, Cochrane Library, and ClinicalTrials.gov was conducted to find eligible studies before May 2021, following Cochrane standards and PRISMA (Preferred Reporting Items for Systematic Reviews and Meta-Analyses) guidelines (28). The language of publication was limited to English and conferences abstract were excluded. The following keywords were used: “endoscopy” or “ureteroscopy” or “lithotripsy” and “obese” or “obesity” or “body mass index” or “BMI” and “urolithiasis” or “calculi” or “stones.” The flowchart is presented in Figure 1. Additional studies were searched by the references of relevant articles.

### Inclusion Criteria and Exclusion Criteria

All eligible articles were selected based upon: (1) researches presenting the outcomes of URS in the OP for urolithiasis and (2) references of included researches were also assessed for eligibility.

The exclusion criteria were: (1) data description was not clear enough; (2) non-English researches; and (3) studies published as review, case reports or comments.

### Data Extraction

The following items were repeatedly extracted by two authors (WW and XSG): author, publication year, country, subgroup, age, number of patients, demographics, stone size, SFR (the definition of SFR was complete stone clearance or presence of residual fragment smaller than 4 mm by ultrasound, CT scan, intravenous pyelography [IVP] or a kidneys-ureters-bladder radiography after the initial procedure), operation time, length of stay, complications based on modified Clavien–Dindo classification system (29). Disagreements were solved by discussing with the third reviewer (XW).

### Quality Assessment

WW and XSG assessed all included studies independently for methodological quality and levels of evidence (LE), and the disagreements were solved by discussion. The Oxford Center for Evidence-Based Medicine (30) was used to estimate the LE of each eligible study. We evaluated the methodological quality of the included reports on the basis of Newcastle Ottawa Scale (31). Additionally, Risk of Bias in Non-randomized Studies-of Interventions (ROBINS-I) was employed to identify the confounding factors for non-randomized studies (32).

### Statistical Analysis

Analysis was performed to utilizing Review Manager Version 5.3 software. Dichotomous data were extracted to utilizing odds ratio (*OR*) and 95% *CI*, and continuous data were extracted by weighted mean difference (MD) and 95% *CI*. The *p*-value was calculated from the *Z*-test, and a *p* ≤ 0.05 was deemed as statistically significant. We assessed the heterogeneity of the included studies by *Q* and *I*^2^ statistics. The *p*-value lower than 0.1 and *I*^2^ value higher than 50% were considered as high heterogeneity among studies, and the random-effects model was performed; if not, indicated low heterogeneity, and the fixed-effects model was conducted. Sensitivity analysis was performed to utilizing a single-item removal method. We utilized a funnel plot to assess publication bias.

## Results

### Study Selection

On the basis of the literature search and the inclusion criteria, 1,208 studies were initially identified. Ultimately, 13 studies (15–27) were included in our analysis, with 4,583 NWP, 2,465 OP, and 291 MOP. Figure 1 shows the literature search and selection process.

### Study Characteristics and Assessment of Quality

Thirteen studies (15–27) were finally included. All were retrospective and the LE of the eligible studies was 3b. Table 1 demonstrated the characteristics of the eligible research. Nine studies (15, 17, 19–22, 25–27) were presented the data regarding stone position, and no statistically difference regarding stone side (*p* = 0.184) and stone location (*p* = 0.061) was observed in the three different populations. There were more category 1 patients of American Society of Anesthesiology (ASA) in the normal weight group relative to the overweight and obese groups (18) (*p* < 0.0001). Predictably, there were more overweight and obese patients categorized as ASA 2 and ASA 3. Therefore, the ASA score is not similar between groups. Additionally, as shown in Table 1, the stone dimension was not different across the groups. Three included studies (16, 20, 21) were provided data regarding stone composition. Best and Nakada reported that nearly 80% of stones consisted of calcium oxalate and uric acid (16). Delorme et al. demonstrated that 64% of stones in OP were calcium oxalate and uric acid (20). Similarly, Doizi et al. revealed that 78% of stones in OP were composed of calcium oxalate and uric acid (21). In all patients, the flexible ureteroscope was advanced up to the kidney after insertion of a ureteral access sheath or guidewire. The outcome parameters of included studies are shown in Table 2. The methodological quality of eligible studies is demonstrated in Supplementary Table 1. The risk of bias of five non-randomized studies is displayed in Supplementary Table 2, and the overall risk of bias judgment of eligible studies was moderate to serious. We performed the forest plots to evaluate the effectiveness of URS for OP.

**Table 1:** Basic characteristics and data of included articles.

**Reference**	**Country**	**Subgroup**	**Patients, n**	**Age, years, mean ±SD**	**M:F**	**BMI, mean ±SD**	**Stone size, mm, mean ±SD**	**Laterality**		**Stone location, n**				**Definition of SFR**
								**R**	**L**	**Renal (Lower pole excluded)**	**Lower pole**	**Ureter**	**Multiple locations**	
Dash et al. (19)	USA	Normal-weight	38	ND	ND	27.3	11 ± 2	ND		23	15			4-week or greater KUB, IVP or CT
		Morbidly obese	16	ND	ND	44.8	13 ± 3	ND		6	10			
Natalin et al. (25)	USA	Normal-weight	34	46.93	9:25	22.74 (17.82–24.85)^a^	9	19	15	1		31		0 to 3 months Post-operatively imaged with CT, and/or IVP
		Overweight	39	51.26	20:19	27.32 (25.10–29.75)^a^	8	24	15	0		38		
		Obese	34	53.32	21:13	33.6 (30.13–45.55)^a^	8.1	19	15	6		28		
Best and Nakada (16)	USA	Non-obese	21	ND	ND	ND	9.7	ND		ND				30-day post-operative imaged with CT, US and KUB
		Obese	22	ND	ND	32.5	8.6	ND		ND				
Delorme et al. (20)	France	Non-obese	149	48.2 ± 1.2	95:54	24 ± 0.2	9.35 ± 0.4	ND		107		29	13	US or CT at 1, 3 and 6 months
		Obese	29	53.5 ± 1.9	14:15	34 ± 0.6	8.38 ± 0.8	ND		23		3	3	
Drăgutescu et al. (23)	Romania	Normal-weight	86	49 (20–79)^a^	45:41	38.8 (35.2–57.7)^a^	9 (5–22)^a^	ND		ND				Post-operatively imaged with CT, KUB, and/or IVP
		Obese	83	47 (22–71)^a^	39:44	22.7 (19.8–24.9)^a^	8 (4–20)^a^	ND		ND				
Caskurlu et al. (17)	Turkey	Normal-weight	81	44.4 ± 13.2	53:28	21.7 ± 1.7	18.6 ± 7.1	36	45	75	53			Day 1 and 1 month post-operatively imaged with KUB and US
		Overweight	77	47.8 ± 13.5	51:26	27.3 ± 0.8	16.9 ± 7.0	33	44	64	47			
		Obese	49	46.6 ± 13.2	31:18	32.8 ± 1.4	18.5 ± 8.4	23	26	45	36			
Chew et al. (18)	Canada and USA	Normal-weight	53	52.2 ± 17.9	ND	22.4 ± 1.8	8.9 ± 0.4	ND		ND				3 months post-operatively imaged with CT, KUB, US, and/or IVP
		Overweight	76	60.9 ± 12.9	ND	27.6 ± 1.3	7.9 ± 0.4	ND		ND				
		Obese	163	52.5 ± 15.4	ND	37.1 ± 7.4	10.4 ± 1.4	ND		ND				
Pompeo et al. (26)	USA	Normal-weight	82	43.8 ± 15.7	46:36	24.2 ± 3.4	9.9 ± 5.0	ND		21	21	40		post-operatively imaged with CT
		Obese	60	47.1 ± 15.8	21:39	33.9 ± 2.1	10.1 ± 7.6	ND		14	23	23		
		Morbidly obese	11	44.5 ± 15.1	2:9	49.0 ± 6.7	13.9 ± 6.5	ND		4	3	4		
Sari et al. (27)	Turkey	Normal-weight	217	40.38 ± 14.3	118:99	ND	164.1 ± 88.3^b^	118	96	70	81	6	60	3 months Post-operatively imaged with CT, KUB and US
		Overweight	162	42.16 ± 15.2	85:77	ND	165.8 ± 92.9^b^	84	74	51	73	3	35	
		Obese	110	40.88 ± 15.2	48:62	ND	185.5 ± 97.9^b^	47 63		23	65	0	22	
		Morbidly obese	13	41.54 ± 16.7	5:8	ND	192.6 ± 85^b^	6 7		1	8	0	4	
Alkan et al. (15)	Turkey	Normal-weight	70	39 ± 12	45:25	22 ± 2	10 ± 6	28	39	ND				2 months post-operatively imaged with CT and US
		Overweight	109	41 ± 11	88:21	27 ± 1	9 ± 5	39	65	ND				
		Obese	77	42 ± 13	50:27	33 ± 2	11 ± 8	28	46	ND				
		Morbidly obese	17	43 ± 16	12:5	44 ± 4	11 ± 8	5	12	ND				
Doizi et al. (21)	France	Normal-weight	188	46.6 ± 15.1	103:85	22.4 ± 2.1	15.2 ± 8.7	106	124	74	92			3 months post-operatively imaged with CT, KUB and US
		Obese	74	53.7 ± 12.6	49:25	34.3 ± 4.6	18.3 ± 13.1	45	52	16	33			
		Morbidly obese	13	51.5 ± 14.1	7:6	43.5 ± 3.3	20.2 ± 13.3	9	5	1	7			
Doluoglu et al. (22)	Turkey	Normal-weight	29	35 (19–84)^a^	21:8	22 (18–24)^a^	15 (5–36)^a^	109	19	17	7		5	1 month post-operatively imaged with KUB and US
		Overweight	49	47 (30–76)^a^	30:19	27 (25–29)^a^	15 (7–35)^a^	19	29	23	14		12	
		Obese	28	51.5(29–84)^a^	10:18	31 (30–40)^a^	14.5 (10–28)^a^	14	14	12	9		7	
Krambeck et al. (24)	USA	Underweight	250	33.69 ± 23.05	107:143	16.91 ± 1.56	55.8 (25.1–117.8)^a^	ND		ND				SFR was determined by post-operative imaging protocols, which differed at each site
		Normal-weight	3,574	45.91 ± 16.66	2,061:1,513	22.65 ± 1.68	51.8 (23.6–94.2)^a^	ND		ND				
		Overweight	4,290	50.58 ± 14.55	3,037:1,253	27.25 ± 1.38	56.5 (31.4–103.7)^a^	ND		ND				
		Obese	1,758	52.56 ± 13.34	1,135:623	32.99 ± 2.51	59.3 (28.3–113.1)^a^	ND		ND				
		Super obese	222	50.61 ± 12.34	91:131	45.58 ± 5.80	55.0 (19.6–117.8)^a^	ND		ND				

a *Using mean (range)*.

b *Using mm^2^*.

*BMI, body mass index; F, female; IVP, intravenous urography; KUB, kidney, ureter, and bladder radiograph; M, male; ND, not documented; SD, standard deviation; SFR, stone-free rate; US, ultrasonography*.

**Table 2:** Outcome parameters of included studies.

**Reference**	**Subgroup**	**Stone-free rate (%)**	**Operation time (minutes)**	**Length of stay (day)**	**Complication rate (%)**
Dash et al. (19)	Normal-weight	84%	ND	ND	36%
	Morbidly obese	67%	ND	ND	14%
Natalin et al. (25)	Normal-weight	91%	70.37 (30–170)	ND	ND
	Overweight	97%	88.78 (30–159)	ND	ND
	Obese	94%	78.23 (30–156)	ND	ND
Best and Nakada (16)	Non-obese	67%	ND	ND	ND
	Obese	82%	ND	ND	ND
Delorme et al. (20)	Non-obese	85%	ND	ND	12%
	Obese	85%	ND	ND	12%
Drăgutescu et al. (23)	Normal-weight	95%	ND	ND	4.5%
	Obese	91%	ND	ND	6.8%
Caskurlu et al. (17)	Normal-weight	79%	49.0 ± 14.6	1.7 ± 1.6	16.0%
	Overweight	77.9%	49.2 ± 14.8	1.8 ± 1.8	15.6%
	Obese	75.5%	52.0 ± 10.3	1.6 ± 1.7	14.3%
Chew et al. (18)	Normal-weight	66%	ND	ND	13%
	Overweight	75%	ND	ND	9%
	Obese	70%	ND	ND	6%
Pompeo et al. (26)	Normal-weight	82.9%	77.5 ± 42.8	ND	ND
	Obese	80%	66.2 ± 35.1	ND	ND
	Morbidly obese	90.9%	85.2 ± 23.4	ND	ND
Sari et al. (27)	Normal-weight	60.8%	ND	1.08 ± 1.06	16.5%
	Overweight	61.7%	ND	0.95 ± 0.51	16.5%
	Obese	73.6%	ND	1.03 ± 0.77	16.3%
	Morbidly obese	61.5%	ND	1.075 ± 0.61	15.4%
Alkan et al. (15)	Normal-weight	81%	60 ± 24	1.08 ± 0.75	16%
	Overweight	87%	67 ± 31	1.04 ± 0.29	13%
	Obese	87.4%	62 ± 29	1 ± 0.375	12%
	Morbidly obese	85%	71 ± 37	1 ± 0.33	15%
Doizi et al. (21)	Normal-weight	67.4%	89.8 ± 42.4	ND	2.6%
	Obese	68%	72.94 ± 42.1	ND	2.1%
	Morbidly obese	71.4%	77.3 ± 43.6	ND	14.3%
Doluoglu et al. (22)	Normal-weight	75.9%	45.5 (10–100)	1 (1–1)	0%
	Overweight	89.8%	50 (30–120)	1 (1–2)	6.1%
	Obese	85.7%	45.5 (25–95)	1 (1–2)	7.1%
Krambeck et al. (24)	Underweight	89.2%	ND	ND	5.6%
	Normal-weight	88.1%	ND	ND	5.0%
	Overweight	87.4%	ND	ND	5.2%
	Obese	83.1%	ND	ND	5.3%
	Super obese	73%	ND	ND	4.9%

*ND, not documented*.

### Stone-Free Rate

**Figure 2:**
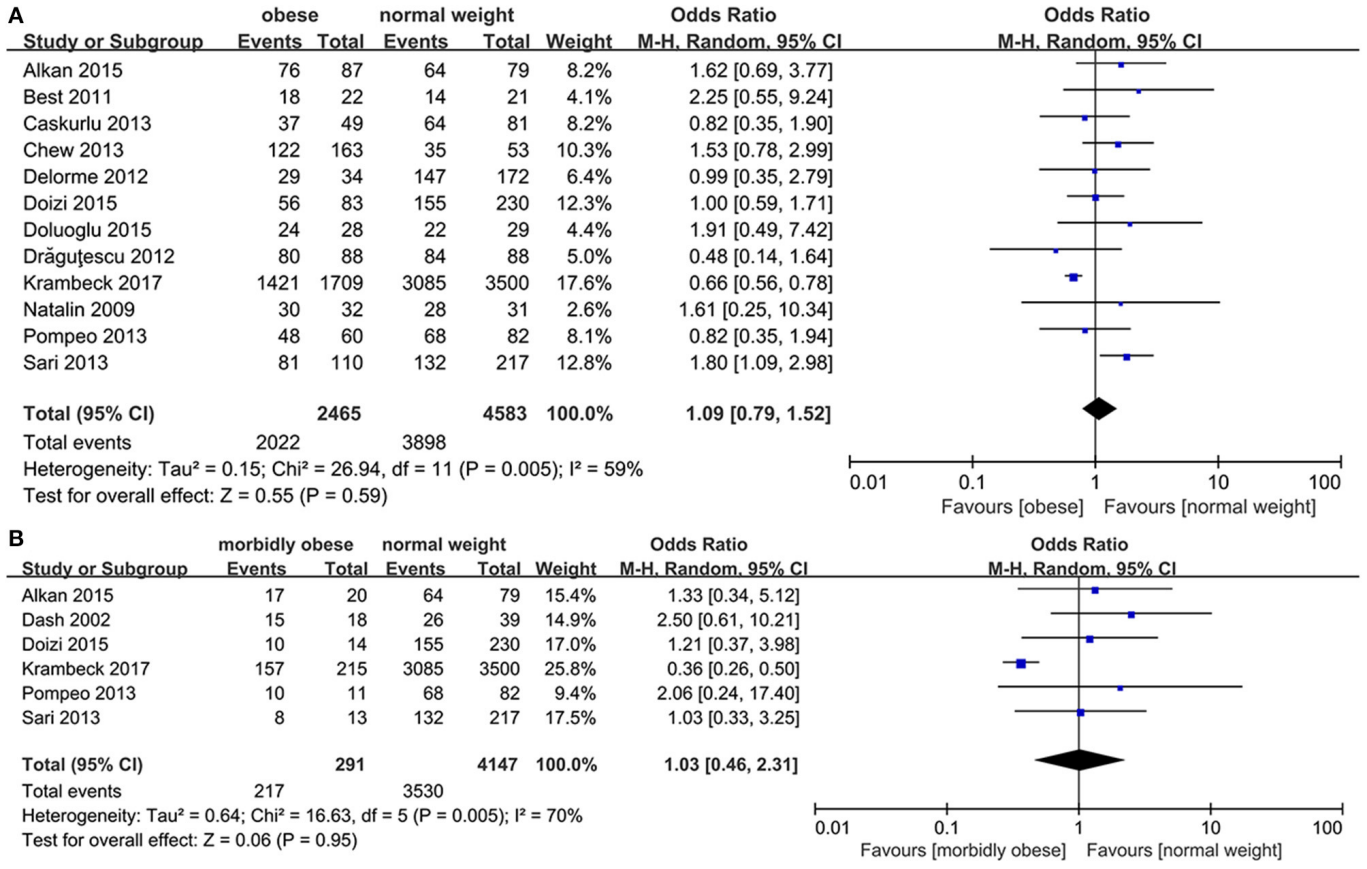
Forest plot of stone-free rate (SFR). **(A)** Obese vs. normal-weight. **(B)** Morbidly obese *vs*. normal-weight.

The 4,583 NWP, 2,465 OP, and 291 MOP were identified. As the heterogeneity was high between OP and NWP (*p* = 0.005, *I*^2^ = 59%) and between MOP and NWP (*p* = 0.005, *I*^2^ = 70%), we used the random-effects model, which indicates no statistically significant difference exist between OP and NWP (*OR*: 1.09; 95% *CI*: 0.79, 1.52; *p* = 0.59), and between MOP and NWP (*OR*: 1.03; 95% *CI*: 0.46, 2.31; *p* = 0.95; Figure 2).

### Operation Time

The three groups were compared concerning operative time. The heterogeneity was high between OP and NWP (*p* = 0.007, *I*^2^ = 68%), and the random-effects model demonstrated that the operation time was similar between two groups (*MD*: −2.27; 95% *CI*: −8.98, 4.43; *p* = 0.51).

**Figure 3:**
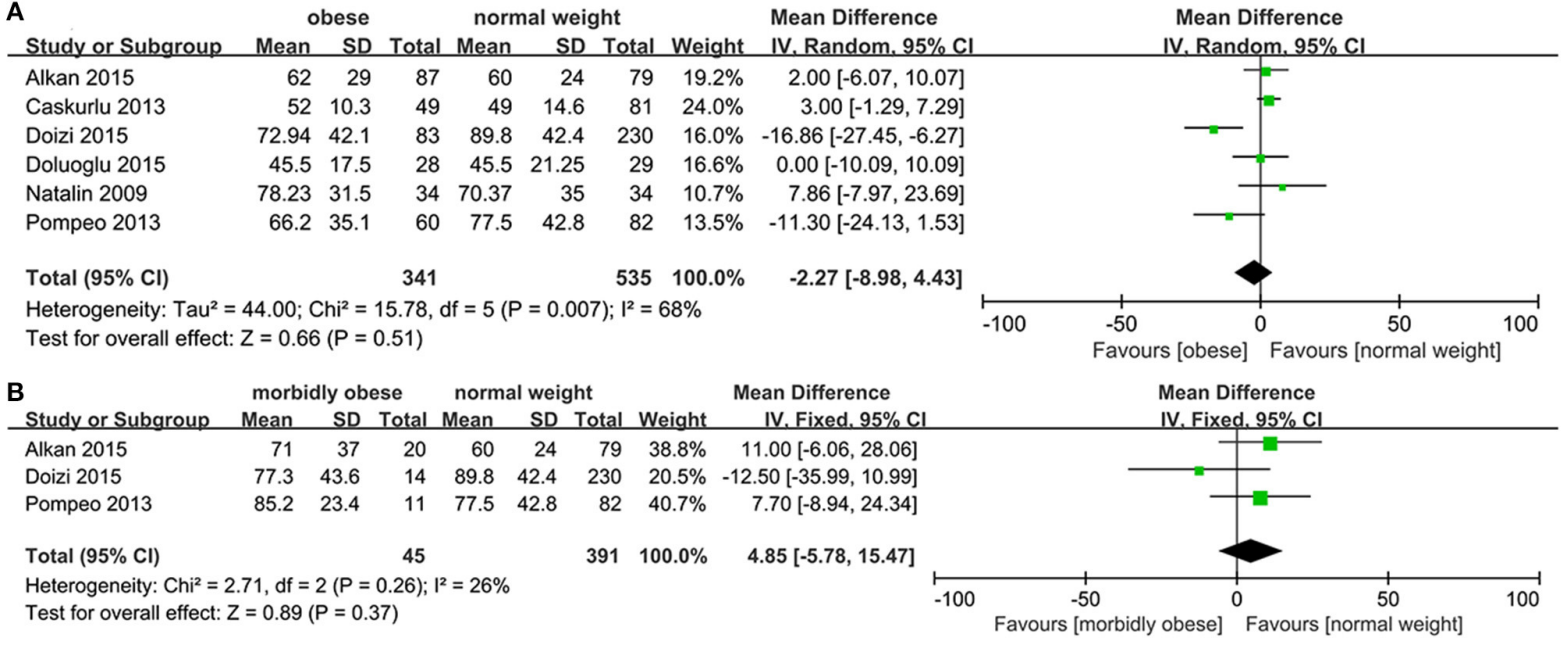
Forest plot of operation time. **(A)** Obese *vs*. normal-weight. **(B)** Morbidly obese *vs*. normal-weight.

As low heterogeneity exists between studies between MOP and NWP, we used the fixed-effects model, which revealed that the operating time was similar between two groups (*MD*: 4.85; 95% *CI*: −5.78, 15.47; *p* = 0.37; Figure 3).

### Length of Stay

**Figure 4:**
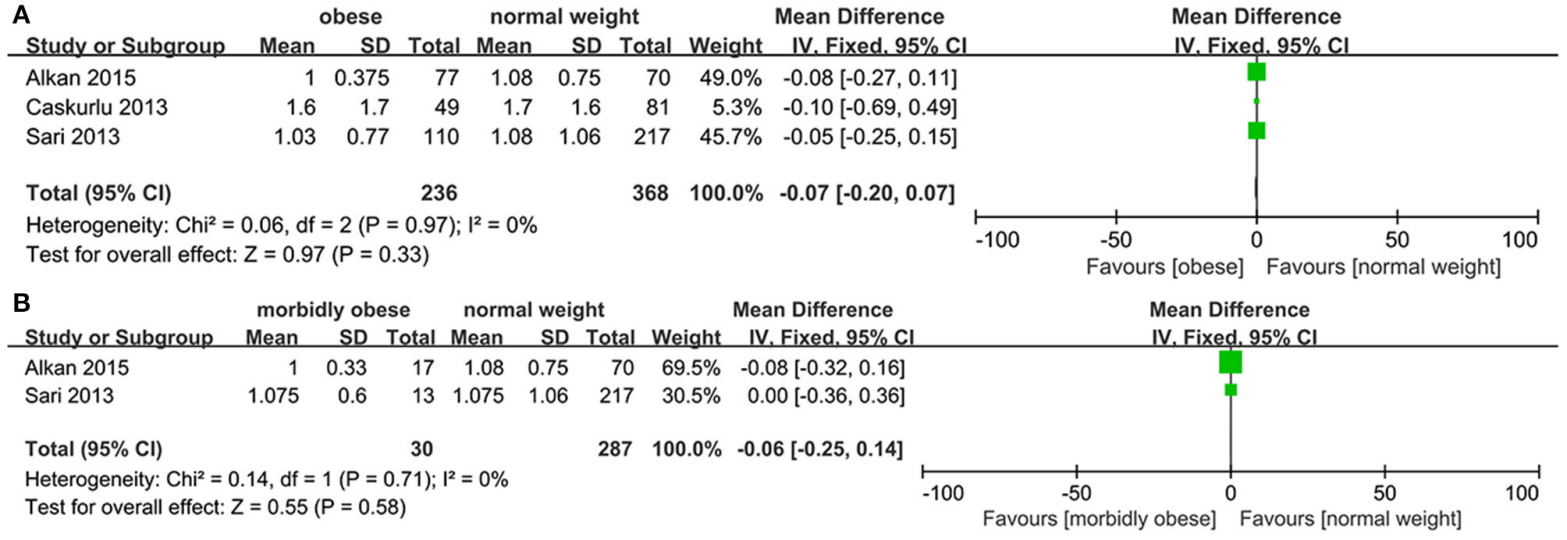
Forest plot of length of stay. **(A)** Obese *vs*. normal-weight. **(B)** Morbidly obese *vs*. normal-weight.

No heterogeneity was seen between OP and NWP (*p* = 0.97, *I*^2^ = 0%), and between MOP and NWP (*p* = 0.71, *I*^2^ = 0%), and the fixed-effects model revealed no statistically significant difference regarding length of stay between OP and NWP (*MD*: −0.07; 95% *CI*: −0.20, 0.07; *p* = 0.33), and between MOP and NWP (*MD*: −0.06; 95% *CI*: −0.25, 0.14; *p* = 0.58; Figure 4).

### Complication Rate

**Figure 5:**
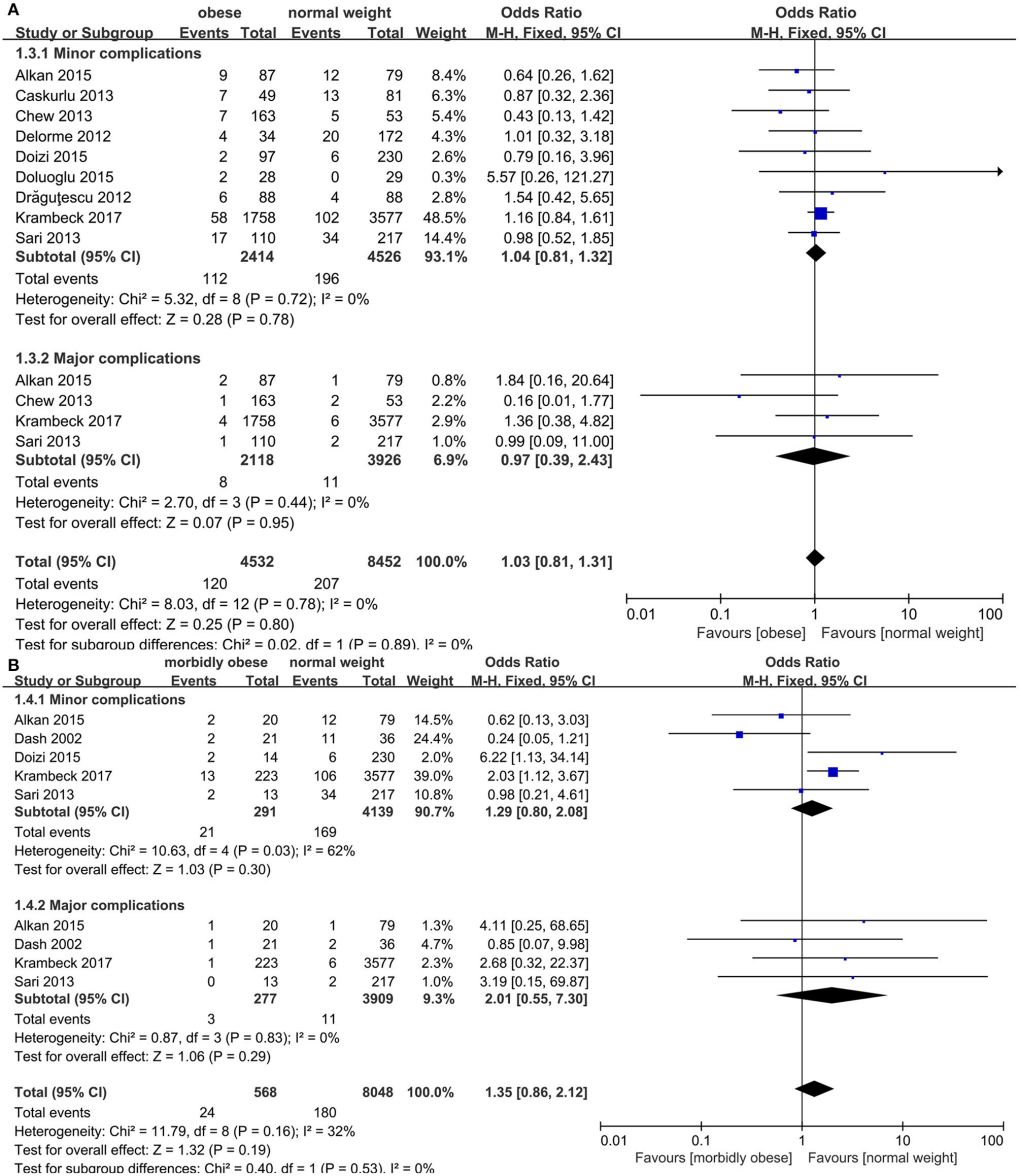
Forest plot of complication rate. **(A)** Obese *vs*. normal-weight. **(B)** Morbidly obese *vs*. normal-weight.

Nine studies (15, 17, 18, 20–24, 27) were provided data regarding the complication rate between OP and NWP. The overall complication rate of MOP and OP was 8.3 and 5.1%, respectively. This is comparable to the complication rate of NWP (4.9%). The pooled result showed that the heterogeneity was low among these studies (*p* = 0.78, *I*^2^ = 0%), and fixed-effects model displayed that no statistically significant difference regarding the overall complication rate were observed between OP and NWP (*OR*: 1.03; 95% *CI*: 0.81, 1.31; *p* = 0.80; Figure 5A). Additionally, no statistically significant difference concerning the minor complications (*OR*: 1.04; 95% *CI*: 0.81, 1.32; *p* = 0.78) and the major complications (*OR*: 0.97; 95% *CI*: 0.39, 2.43; *p* = 0.95; Figure 5A) were found between OP and NWP.

Five studies (15, 19, 21, 24, 27) were providing data in terms of the complication rate among MOP and NWP. The heterogeneity was low (*p* = 0.16, *I*^2^ = 32%) and fixed-effect model revealed that the overall complication rate was similar between two groups (*OR*: 1.35; 95% *CI*: 0.86, 2.12; *p* = 0.19; Figure 5B). Moreover, no statistically significant difference concerning the minor complications (*OR*: 1.29; 95% *CI*: 0.80, 2.08; *p* = 0.30) and the major complications (*OR*: 2.01; 95% *CI*: 0.55, 7.30; *p* = 0.29; Figure 5B) were seen between MOP and NWP.

### Sensitivity Analysis and Publication Bias

**Figure 6:**
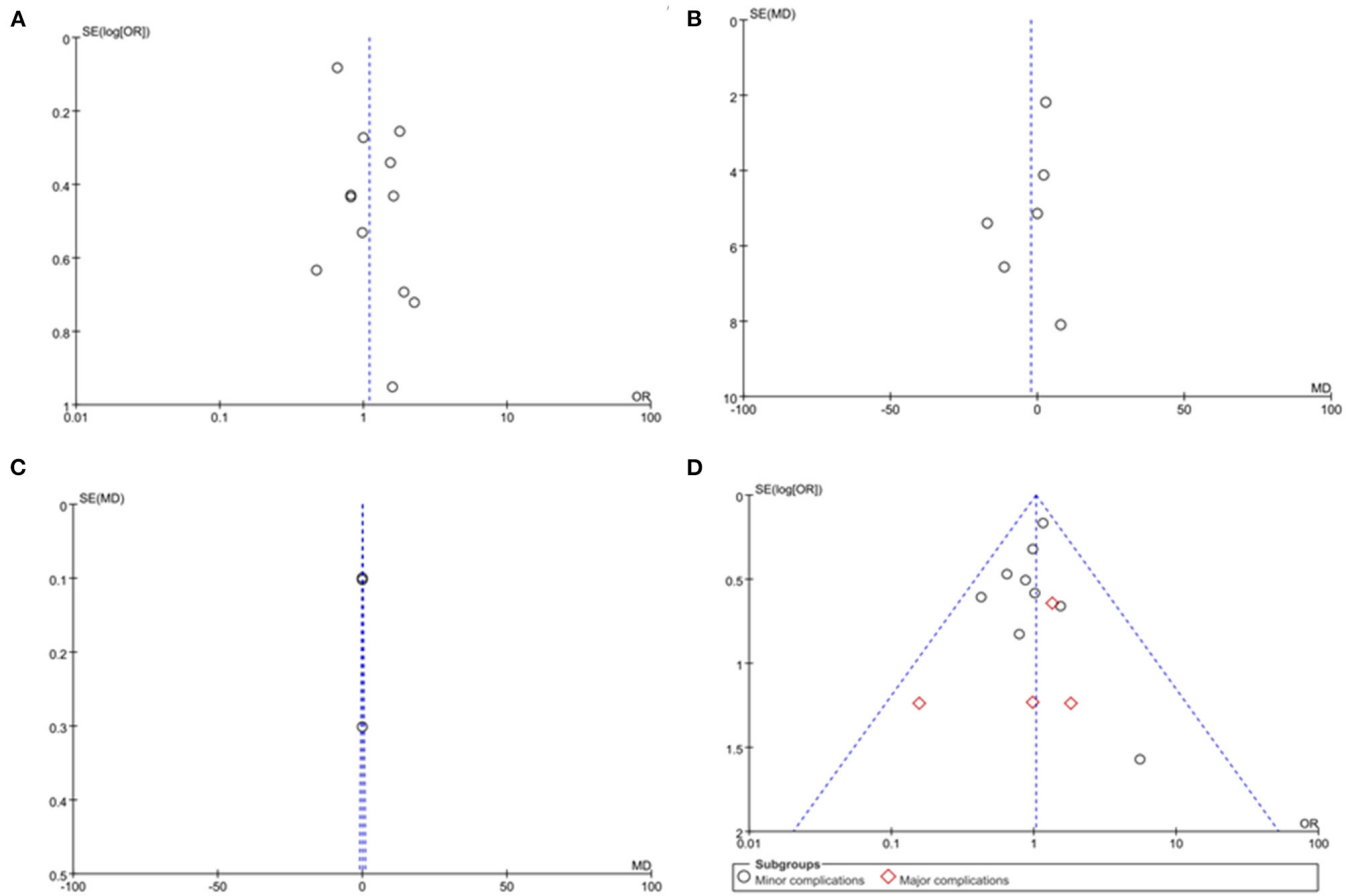
Funnel plot evaluating the publication bias. **(A)** Stone-free rate. **(B)** Operation time. **(C)** Length of stay. **(D)** Complication rate.

Sensitivity analysis was performed and demonstrated the stable results. When it was applied to SFR between OP and NWP, and between MOP and NWP, one study (24) was the main source for high heterogeneity; after removal of the study, no heterogeneity was seen and the results did not change. Similarly, one study (21) was the main reason for the high heterogeneity of operative time between OP and NWP; after excluding the study, the heterogeneity was declined significantly and the results were remained unchanged. We utilized a funnel plot to estimate publication bias, and the symmetrical distribution indicated no obvious existence of publication bias (Figure 6).

## Discussion

The worldwide incidence of obesity is rapidly growing during the last decades. It is estimated that the number of OP grows from 400 million in 2005 to 700 million in 2015 (33). Obesity is related to many health problems such as diabetes mellitus (2), hypertension (3), cardiomyopathy (4), and prostate cancers (5). It is also well-documented that there exist is an association between obesity and a higher risk of urolithiasis (34). Scales et al. uncovered that the prevalence of urolithiasis in obese and normal-weight individuals was 11.2 and 6.1%, respectively (35). Studies reported that stones in OP mainly consisted of calcium oxalate and uric acid (36), which is consistent with our analysis. Insulin resistance caused by obesity might be the most important factor associated with urolithiasis. Strohmaier et al. reported that insulin resistance decreased the production and transportation of ammonia, leading to a low urine pH (37), whereas an acid urine pH facilitated the formation of uric acid stones (34). In contrast, calcium oxalate stones, as the most common composition of stones in OP, are hardly influenced by obesity. When faced with the OP with urolithiasis, urologists are likely to stick in a dilemma of how to manage these individuals because the removal of stones is risky and difficult. Due to cardiovascular, respiratory, and metabolic alteration, obese individuals are susceptible to perioperative comorbidities and increase morbidity (13). Therefore, it is recommended to use mini-invasive procedures rather than open surgery in obese individuals.

Technological development in endoscope equipment has prompted the emergence of smaller scope with a better angle of deflection and laser technology, which renders more efficacious stone fragmentation while retaining hemostasis. All these make URS an attractive procedure to manage urolithiasis (38). In our study, we comprehensively assessed the effectiveness and safety of URS on obese individuals with normal weight persons controlled. We noticed that no statistically significant difference existed between OP and NWP, and between MOP and NWP groups regarding SFR, complications, operation time, and length of stay, indicating that URS performed on OP and MOP is equally secure and efficacious to NWP.

Stone-free rate is a critical outcome variable to assess the effectiveness of endourological surgery. Chew et al. reported that MOP was associated with a relatively lower SFR compared with NWP (18). However, only eight MOPs were enrolled in their study. In addition, a study published by Krambeck et al., includes 11,885 patients concluded that the obesity was related to lower SFR and the efficacy of URS procedure was decreased compared with normal weight group (24). But the median stone burden in this study exceeded 50 mm in each group, which might significantly affect the SFR of URS procedure. Furthermore, the authors utilized the results of a multicenter study, in which SFR status differed at each site and was not clearly clarified (24). The reason why obesity has no influence on SFR in our analysis might be that the external fat tissue has no necessary connection with abnormal internal anatomy. In addition, URS procedure is performed by inserting the scope into the ureter, and this process is not influenced by increased retroperitoneal fat tissue. Specially, two included studies (19, 21) presented SFR in patients whose stone sizes were <10 mm. Dash et al. reported that the success rates for stones <10 mm were 100 and 69% in MOP and NWP groups, respectively. However, they did not observe a significant difference between two groups (*p* = 0.43). Similarly, Doizi et al. found that no significant differences regarding SFR for stones <10 mm existed between OP and NWP, and between MOP and NWP (93% SFR in NWP, 100% SFR in OP, and 100% SFR in MOP).

No statistically differences existed in operation time and length of stay between OP and NWP, and between MOP and NWP groups in our study. Ishii et al. conducted a meta-analysis and demonstrated that the mean operation time for the OP and MOP was 68.5 and 68.9 min, respectively (39). In another study by Alkan et al. revealed that the mean hospital stay for the OP and MOP was 1 ± 0.375 and 1 ± 0.33 day, respectively. Similarly, no statistically significant difference existed between OP and NWP, and between MOP and NWP groups regarding operation time and length of stay in our analysis, indicating that URS performed in MOP and OP appear to have the same safety as well as in the NWP group.

In terms of post-operative morbidity, we assessed the complication rate between the different groups and no statistically significant difference was observed. In our analysis, the overall complication rate of MOP and OP was 8.3 and 5.1%, respectively. This is comparable to the complication rate of NWP (4.9%). Ishii et al. reported that the complication rate in the OP was 8.4%, which is consistent with our results. But they only observed 16 complications in MOP (17.6%) (39). They concluded that the complication rate tended to be higher in MOP. The reason for the relatively higher complication rate in their study might be that only 91 MOP was included in the analysis so the results were inaccurate and biased. Additionally, 5 included studies (15–17, 19, 22) followed their patients up to 1 month post-operatively, and 5 included studies (18, 20, 21, 24, 27) saw their patients after 3 months in our analysis. Other authors did not specify the follow-up interval. The different time period of follow-up of included study might also contribute to distinguishing complication rate. However, the majority of complications were Clavien I or II, and no death occurred in our analysis, which corresponds to the previous study (39). Therefore, we can conclude that URS performed on OP and MOP is equally safe to NWP.

Life style modification is the most important method for the prevention of recurrence of urolithiasis in OP (40). A diet containing a variety of fruits, vegetables, whole grains, and low-fat food is beneficial to OP (40). Besides, the combination of healthy diet and exercise will benefit patients more effective than diet or exercise alone (40).

A previous systematic review that included 7 studies involving 131 individuals found that URS could be conducted effectively and safely on OP, and SFR increased when stones size was <20 mm (14). However, this study was the small sample sized and did not sub-categorize the findings into obese and morbidly obese groups. Another systematic review demonstrated that URS was a secure and efficacious treatment choice for OP (39). Nonetheless, the authors failed to assess the effectiveness and safety of obesity on URS with a matched group of normal-weight individuals. They compared the outcomes of URS in the OP and MOP, concluding that the complications tended to be higher in MOP (39). However, the overall complication rate in MOP (8.3%) was close to OP (5.1%), and both were comparable to NWP (4.9%) in our analysis. In addition, the number of patients in our analysis was around 3 times (2465 OP and 291 MOP) more compared with the previous study (39).

Additionally, several limitations need to be mentioned in the present analysis. First, all the eligible studies were retrospective, which are associated with methodology bias. Second, the definition of SFR was not standardized, several studies considered residual stones ranging from 0 to 4 mm as stone-free status (15–17, 19, 21), while two studies only defined SFR as no fragments of any size (18, 20). In addition, there were various radiological methods for determining SFR in the included studies, such as ultrasound, CT scan, and IVP, and without a good clarification that which one is the best technique. Next, considering the patient positioning issue, the total operative room time should also be evaluated in addition to procedure time. But none of our included studies presented this data. Future studies providing more complete information are needed. However, our analysis incorporated a group of NWP as controlled and is the largest sample sized studies that determined the URS outcome variables in OP and MOP.

## Conclusion

The URS procedure demonstrated similar results in MOP, OP, and NWP. Therefore, URS performed in MOP and OP appears to have the same efficacy and safety as well as in NWP group.

## Data Availability Statement

The original contributions presented in the study are included in the article/Supplementary Material, further inquiries can be directed to the corresponding author/s.

## Author Contributions

WW and TJ collected and analyzed the data and prepared the manuscript. XG collected the data. LP analyzed the data and prepared a manuscript. All authors have read and approved the final manuscript.

## Conflict of Interest

The authors declare that the research was conducted in the absence of any commercial or financial relationships that could be construed as a potential conflict of interest.

## Publisher's Note

All claims expressed in this article are solely those of the authors and do not necessarily represent those of their affiliated organizations, or those of the publisher, the editors and the reviewers. Any product that may be evaluated in this article, or claim that may be made by its manufacturer, is not guaranteed or endorsed by the publisher.

## Abbreviations

URS: ureteroscopy
BMI: body mass index
SFR: stone-free rate
OR: odds ratio
MD: weighted mean difference
IVP: intravenous pyelography
LE: levels of evidence
NOS: Newcastle Ottawa Scale.
