# Supplementary material for: Ureteroscopy Is Equally Efficient and Safe in Obese and Morbidly Obese Patients: A Systematic Review and Meta-Analysis
Source: Front Surg. 2022 Feb 18;9:736641. doi: 10.3389/fsurg.2022.736641 (PMC8894321; doi:10.3389/fsurg.2022.736641)
Supplement: Supplementary file 1 [file Table_1.DOCX]

Supplementary table 1. Methodological quality of included 13 studies.

|  | Definition adequate | Representativeness | Selection of Controls | Definition of Controls | Comparability of age | Other controlled factor | Ascertainment of exposure | Same method of ascertainment | Non-Response Rate | Total score |
| --- | --- | --- | --- | --- | --- | --- | --- | --- | --- | --- |
| Dash et al. | ★ | ★ |  | ★ |  |  | ★ | ★ | ★ | 6 |
| Natalin et al. | ★ | ★ |  | ★ |  |  | ★ | ★ | ★ | 6 |
| Best et al. | ★ | ★ |  | ★ |  |  | ★ | ★ |  | 5 |
| Delorme et al. | ★ | ★ |  | ★ | ★ | ★ | ★ | ★ | ★ | 8 |
| Drăguţescu et al. | ★ | ★ |  | ★ |  |  | ★ | ★ |  | 5 |
| Caskurlu et al. | ★ | ★ |  | ★ |  |  | ★ | ★ | ★ | 6 |
| Chew et al. | ★ | ★ |  | ★ | ★ | ★ | ★ | ★ | ★ | 8 |
| Pompeo et al. | ★ | ★ |  | ★ | ★ | ★ | ★ | ★ | ★ | 8 |
| Sari et al. | ★ | ★ |  | ★ | ★ | ★ | ★ | ★ | ★ | 8 |
| Alkan et al. | ★ | ★ |  | ★ | ★ | ★ | ★ | ★ | ★ | 8 |
| Doizi et al. | ★ | ★ |  | ★ |  |  | ★ | ★ | ★ | 6 |
| Doluoglu et al. | ★ | ★ |  | ★ |  |  | ★ | ★ | ★ | 6 |
| Krambeck et al. | ★ | ★ |  | ★ |  |  | ★ | ★ | ★ | 6 |
