# Supplementary material for: Ureteroscopy Is Equally Efficient and Safe in Obese and Morbidly Obese Patients: A Systematic Review and Meta-Analysis
Source: Front Surg. 2022 Feb 18;9:736641. doi: 10.3389/fsurg.2022.736641 (PMC8894321; doi:10.3389/fsurg.2022.736641)
Supplement: Supplementary file 2 [file Table_2.DOCX]

*Supplementary Table 2*. Result of Risk of Bias (RoB) Assessment of studies *^a^*

| Sources | Risk of Bias Judgement for Domain | | | | | | | Overall RoB Judgement of the Result |
| --- | --- | --- | --- | --- | --- | --- | --- | --- |
|  | Bias due to confounding | Bias in the selection of participants in the study | Bias in the classification of interventions | Bias due to deviations from intended interventions | Bias due to missing data | Bias in measurement of outcomes | Bias in selection of reported result |  |
| Dash 2002 | No Information | Low | Moderate | No Information | Moderate | Serious | Moderate | Serious |
| Natalin 2009 | Moderate | Low | Low | Serious | Serious | Serious | Moderate | Serious |
| Best 2011 | Serious | Low | Low | Low | Moderate | Serious | Low | Serious |
| Delorme 2012 | Serious | Low | Serious | No Information | Serious | Serious | Moderate | Serious |
| Drăguţescu 2012 | Serious | Low | Serious | Low | No information | Low | Moderate | Serious |
| Caskurlu 2013 | Low | Low | Low | Low | Low | Serious | Moderate | Serious |
| Chew 2013 | Moderate | Low | Low | Moderate | Moderate | Low | Moderate | Moderate |
| Pompeo 2013 | Serious | Low | Serious | No information | No information | Serious | Low | Serious |
| Sari 2013 | Low | Low | Low | No information | Low | Moderate | Moderate | Moderate |
| Alkan 2015 | Low | Low | Low | No information | Moderate | Moderate | Low | Moderate |
| Doizi 2015 | Low | Low | Low | Moderate | Moderate | Moderate | Low | Moderate |
| Doluoglu 2015 | Low | Low | Low | Moderate | Low | Moderate | Low | Moderate |
| Krambeck 2017 | Serious | Low | Low | Moderate | Moderate | Serious | Serious | Serious |

*Note: ^a^* = Based on assessment using ROBINS-I tool by Sterne JAC, Hernán MA, Reeves BC, Savović J, Berkman ND, Viswanathan M, Henry D, Altman DG, Ansari MT, Boutron I, Carpenter JR, Chan AW, Churchill R, Deeks JJ, Hróbjartsson A, Kirkham J, Jüni P, Loke YK, Pigott TD, Ramsay CR, Regidor D, Rothstein HR, Sandhu L, Santaguida PL, Schünemann HJ, Shea B, Shrier I, Tugwell P, Turner L, Valentine JC, Waddington H, Waters E, Wells GA, Whiting PF, Higgins JPT. ROBINS-I: a tool for assessing risk of bias in non-randomized studies of interventions*. BMJ* 2016; 355; i4919.
